# A Communication-Efficient Distributed Matrix Multiplication Scheme with Privacy, Security, and Resiliency

**DOI:** 10.3390/e26090743

**Published:** 2024-08-30

**Authors:** Tao Wang, Zhiping Shi, Juan Yang, Sha Liu

**Affiliations:** 1 National Key Laboratory of Wireless Communications, University of Electronic Science and Technology of China, Chengdu 611731, China; 202211220615@std.uestc.edu.cn (T.W.); shaliu514@163.com (S.L.); 2 School of Electronic Information and Automation, Guilin University of Aerospace Technology, Guilin 541004, China; yj13978357370@outlook.com; 3 Guangxi Key Laboratory of Automatic Detecting Technology and Instruments, Guilin University of Electronic Technology, Guilin 541004, China

**Keywords:** secure distributed matrix multiplication, trace polynomials, Reed–Solomon codes, interleaved codes

## Abstract

Secure distributed matrix multiplication (SDMM) schemes are crucial for distributed learning algorithms where extensive data computation is distributed across multiple servers. Inspired by the application of repairing Reed–Solomon (RS) codes in distributed storage and secret sharing, we propose SDMM schemes with reduced communication overhead through the use of trace polynomials. Specifically, these schemes are designed to address three critical concerns: (i) ensuring information-theoretic privacy against collusion among servers; (ii) providing security against Byzantine servers; and (iii) offering resiliency against stragglers to mitigate computing delays. To the best of our knowledge, security and resiliency are being considered for the first time within trace polynomial-based approaches. Furthermore, our schemes offer the advantage of reduced sub-packetization and a lower server-count requirement, which diminish the computational complexity and download cost for the user.

## 1. Introduction

Large-scale matrix multiplication is a fundamental building block in today’s big data era, finding applications in various fields such as signal processing, wireless communication, and machine learning. With the increasing scale of large language models, computing on a single server is no longer feasible. Therefore, distributing large matrix computation tasks across multiple servers becomes a viable solution. In distributed computing, when all servers are considered trustworthy, the increased computation time primarily stems from two factors: (i) the need to await responses from slower servers (stragglers), and (ii) network congestion resulting from the transmission of large volumes of data. Furthermore, the situation becomes more complex when some servers are untrustworthy, especially if the computation includes sensitive information. In such a scenario, servers might share their received data to infer the contents of original matrices held by the user, which compromises the privacy of the user. This highlights the importance of researching communication-efficient SDMM schemes.

Polynomial codes were initially proposed by Yu et al. in [1] to mitigate the impact of stragglers on computation time in distributed matrix multiplication. Subsequent works [2,3,4,5,6] extended Yu et al.’s work by incorporating random matrices during the upload phase of polynomial codes, constructing information-theoretically secure SDMM schemes that meet specific privacy requirements with a minimal number of servers. Different schemes have also been introduced in [7,8,9,10]. The above efforts concern the scenario in which servers are untrustworthy. More generally, they are concerned with scenarios when servers are considered untrustworthy-but-useful, implying that some might deviate from the predefined algorithms. In this case, the system may not only have colluding servers but also Byzantine servers—those who intentionally or erroneously return incorrect results to the user for their own benefit, significantly impacting SDMM by yielding erroneous computation results and rendering the efforts of normal servers futile. An SDMM scheme is said to provide security against Byzantine servers if it can ensure the correctness of computation results even in the presence of Byzantine servers within the system. There are several schemes proposed in the literature [11,12,13] that address the challenges posed by stragglers and Byzantine servers. These schemes treat stragglers as erasures and Byzantine servers as errors within some linear codes. As a result, handling a straggling server requires one additional server, whereas addressing a Byzantine server necessitates two additional servers.

Repairing RS codes were first introduced in distributed storage systems by Guruswami et al. in [14], aiming to repair a failed node with lower communication overhead. It was further extended in [15] to address the repair problem of multiple failed nodes. In such systems, a large file is encoded into fragments before being distributed across multiple nodes. When a node fails, the system initiates a replacement node, which is required to recover the data stored at the failed node by receiving the necessary information from the remaining surviving nodes (helper nodes). The repair bandwidth is the total volume of data (in bits) required by the replacement node during the repair process. To minimize the repair bandwidth, the approach proposed in [14] utilizes trace polynomials, enabling the helper nodes to transmit only part of their contents to the replacement node while still ensuring precise data recovery. This approach involves breaking up a symbol in $F_{q}^{t}$ into *t* symbols in $F_{q}$, where *t* is referred to as sub-packetization. In order to recover the data in a failed node with as little repair bandwidth as possible, the repairing RS codes constructed in [16] are designed to meet the cut-set bound. It is worth noting that, as discussed in [15], SDMM is similar to repairing multiple failed nodes in a distributed storage system, where servers are analogous to helper nodes and the user is analogous to the replacement node.

Inspired by the work in [16], Machado et al. were the first to employ the field trace function in polynomial codes (FTP), constructing a communication-efficient fully *X*-private SDMM scheme [17]. This scheme ensures that the user can securely compute matrix multiplication MN using $d=p_{L}+2L+2X-2$ servers without revealing any information about the matrices $M\in F_{q^{t}}^{a\times b}$ and $N\in F_{q^{t}}^{b\times c}$, even in the presence of up to *X* colluding servers. In order to meet the cut-set bound, this scheme possesses sub-packetization $t=p_{1}p_{2}\cdots p_{L}$, where *L* denotes the number of blocks in *M*, $\left\{p_{1},p_{2},\cdots ,p_{L}\right\}$ is a set of prime numbers in increasing order, and *q* is a prime power. It is widely recognized that minimizing sub-packetization is essential, as the time complexity of multiplication increases significantly with the size of the finite field. In [18], schemes based on trace polynomials and subspace polynomials for computing linear combinations of coded symbols were introduced. These schemes provide information-theoretic privacy for either *M* or *N* amidst *X* colluding servers, with sub-packetization $t\geq \log_{q}\left(\frac{L+X-1}{q^{s}}+L\right)+s$, under the condition $q^{t-s}>L$, where *s* is the dimension of the subspace. In many cases, the sub-packetizations in these schemes are smaller than those in [17]. It is noteworthy that the schemes in [18] are more suitable for distributed computation scenarios compared to FTP codes, as the user can directly obtain the desired value, while FTP codes require additional operations. However, the schemes outlined in [18] necessitate at least $d\geq Lq^{s}+X-1$ servers, a requirement that poses challenges for practical implementation. Moreover, neither of the aforementioned schemes considered the robustness of SDMM against Byzantine servers and stragglers.

The comparison between our schemes and previous works is shown in Table 1 and discussed in more detail in Section 4 and  Section 5. Table 1 presents a comparison of FTP codes [17], the scheme proposed in [18], and our proposed schemes in terms of key parameters. Note that in Table 1, the header “Download cost” indicates the number of symbols from $F_{q}$ that the user needs to download from the servers in the corresponding scheme. The header “Remark” specifies whether the fully *X*-privacy SDMM scheme is robust against Byzantine servers and stragglers.

Building on the discussions above, this paper proposes communication-efficient SDMM schemes that provide privacy against colluding servers, security against Byzantine servers, and resiliency against stragglers. Specifically, (i) in Section 4, the minimal polynomial is utilized to construct communication-efficient one-sided and fully *X*-privacy SDMM schemes; (ii) in Section 5, by refining the data returned by the servers to form an *L*-interleaved code, ensuring that our schemes provide security against Byzantine servers; (iii) by treating the impact of stragglers as erasures in linear codes, our schemes also demonstrate resiliency against straggler servers.

## 2. System Model and Problem Formulation

We consider a scenario where a user possesses matrices $M\in F_{q^{t}}^{a\times b}$ and $N\in F_{q^{t}}^{b\times c}$ and intends to securely compute their product MN with the collaborative effort of *d* servers. The matrices *M* and *N* are respectively partitioned by columns and rows into *L* blocks, as follows:

$$M=\left[\begin{array}{cccc} M_{1} & M_{2} & \cdots & M_{L} \end{array}\right],\;N=\left[\begin{array}{c} N_{1}\\ N_{2}\\ \vdots \\ N_{L} \end{array}\right],$$

then $MN=M_{1}N_{1}+M_{2}N_{2}+\cdots +M_{L}N_{L}$. It can be observed that computing the product MN is equivalent to computing $M_{i}N_{i}$ for all i∈{1,2,⋯,L}. The user connects to each server through a private link and assumes that these servers are untrustworthy-but-useful. To prevent any leakage of information about *M* and *N* to the servers, the user sends the securely encoded versions $m_{j}$ and $n_{j}$ of *M* and *N* to each server *j*, for j∈{1,2,⋯,d}. Upon receiving the encoded matrices, server *j* computes the response $E_{j}$ such that $MN=decode(E_{1},E_{2},\cdots ,E_{d})$, where decode is noted as the decoding function.

**Figure 1:**
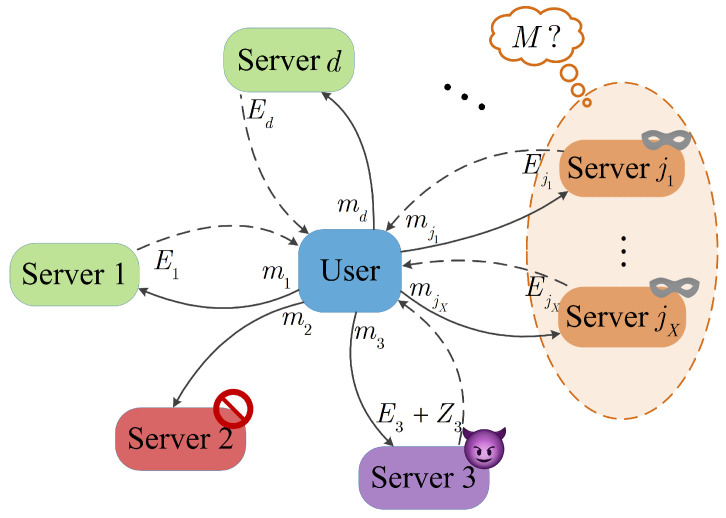
System model of one-sided SDMM framework: green boxes represent normal servers, yellow boxes represent colluding servers, red boxes represent straggle servers, and purple boxes represent Byzantine servers.

One of the key requirements in SDMM is to provide information-theoretic privacy for the matrices that the user possesses, even in the presence of *X* colluding servers. Information-theoretic privacy means that no matter how powerful the computational capabilities of these colluding servers are, they cannot gain any information about the matrices owned by the user. In this paper, we first study the one-sided privacy SDMM scheme with *X* colluding servers. In this scenario, matrix *N* is accessible to all servers, while matrix *M* is exclusively owned by the user. The primary objective is to securely compute MN without disclosing any information about *M* to any *X* colluding servers. Moreover, the user is unaware of which *X* servers may potentially collude. As shown in Figure 1, let $𝒳=\left\{j_{1},j_{2},\cdots ,j_{X}\right\}\subset \left\{1,2,\cdots ,d\right\}$ be the index set of *X* colluding servers, and $m_{𝒳}≜\left\{m_{j_{1}},m_{j_{2}},\cdots ,m_{j_{X}}\right\}$. These colluding servers intend to infer the contents of *M* from $m_{𝒳}$. To achieve information-theoretical *X*-privacy in a scheme, the encoded fragments $m_{𝒳},\;\forall 𝒳\subset \left\{1,2,\cdots ,d\right\},\;\left|𝒳\right|=X$ must not leak any information about *M*, i.e.,

$$I\left(M;m_{𝒳}\right)=0,\;\forall 𝒳\subset \left\{1,2,\cdots ,d\right\},\;\left|𝒳\right|=X.$$

When both *M* and *N* are exclusively owned by the user, a scheme that securely computes MN without disclosing any information about *M* and *N* to any *X* colluding servers is referred to as information-theoretically fully *X*-privacy. Let $𝒳=\left\{j_{1},j_{2},\cdots ,j_{X}\right\}\subset \left\{1,2,\cdots ,d\right\}$ denote the set of indices for the *X* colluding servers. In this case, $m_{𝒳}≜\left\{m_{j_{1}},m_{j_{2}},\cdots ,m_{j_{X}}\right\}$ and $n_{𝒳}≜\left\{n_{j_{1}},n_{j_{2}},\cdots ,n_{j_{X}}\right\}$ should not leak any information about *M* and *N*. The privacy constraint in this case is

$$I\left(M,N;m_{𝒳},n_{𝒳}\right)=0,\;\forall 𝒳\subset \left\{1,2,\cdots ,d\right\},\;\left|𝒳\right|=X.$$

One of the bottlenecks in distributed computing is network congestion caused by the transmission of large amounts of data during computation. Therefore, it is crucial to study matrix multiplication schemes with communication efficiency. This paper proposes a communication-efficient SDMM scheme that differs from traditional polynomial-based schemes. Let E be a *t*-degree extension field of a finite field F. We incorporate the concept of repairing Reed–Solomon codes from distributed storage into distributed matrix multiplication. Specifically, each server *j* stores the evaluation of the polynomial h(x)∈E[x] at point $\alpha_{j}\in E$, where MN corresponds to the data stored on failed nodes. By carefully designing h(x), a distributed matrix multiplication scheme that ensures *X*-privacy can be constructed. To reduce the amount of data transmitted over the network, the key tool in designing the communication-efficient SDMM scheme is the trace function. The trace function $Tr_{E/F}:E\to F$ maps elements from E to F. Specifically, the trace function $Tr_{E/F}$ maps a *t*-dimensional vector over F to an element in F. In summary, in the SDMM scheme proposed in this paper, server *j* does not send the full evaluation $h(\alpha_{j})$, but instead sends $Tr_{E/F}\left(h\left(\alpha_{j}\right)\right)$ to help the user compute MN, thereby reducing the amount of data transmitted in SDMM. Since the servers are considered untrustworthy-but-useful, the system may not only have colluding servers but also possible Byzantine servers who might return incorrect computation results $E_{j}+Z_{j}$ to the user, for some non-zero $Z_{j}$. Additionally, there may be stragglers in the system who fail to return the computation results on time. In Figure 1, servers 3 and 2 represent these two types of servers, respectively. Hence, when designing an SDMM scheme, multiple goals must to be considered, including reducing communication overhead, providing security against Byzantine servers, and mitigating the impact of stragglers on computation time.

Coding theory tools are employed to design a communication-efficient SDMM scheme that meets the aforementioned objectives. Specifically, as discussed previously, when the data returned by all servers forms a linear code, these tools can correct errors introduced by Byzantine servers and mitigate erasures caused by stragglers.

## 3. Preliminaries

Some notation and essential concepts are introduced in this section. In the rest of this paper, we write [n]={1,2,⋯,n}.

**Definition** **1.** *Let q be a prime power, $F_{q}\left[x\right]$ be the polynomial ring over finite field $F_{q}$. An RS code RS(Ω,k) over $F_{q}$ is defined as*

$$RS\left(\Omega ,k\right)=\left\{{\left(f(\alpha )\right)}_{\alpha \in \Omega }:f\left(x\right)\in F_{q}\left[x\right],\;\deg\left(f(x)\right)<k\right\},$$

*where $\Omega =\{\alpha_{1},\alpha_{2},\cdots ,\alpha_{n}\}$ and n>k.*

The dual code of RS(Ω,k) is a generalized RS code

$$GRS\left(\Omega ,n-k,V\right)=\left\{{\left(vh(\alpha )\right)}_{v\in V,\alpha \in \Omega }:h\left(x\right)\in F_{q}\left[x\right],\;\deg\left(h(x)\right)<n-k\right\},$$

where $V=\{v_{1},v_{2},\cdots ,v_{n}\}$ is determined by Ω:

(1) $$v_{i}=\prod_{m\in [n],m\neq i}{\left(\alpha_{i}-\alpha_{m}\right)}^{-1}.$$

It is evident that

(2) $$\sum_{i\in [n]}f\left(\alpha_{i}\right)v_{i}h\left(\alpha_{i}\right)=0.$$

**Definition** **2.** *Let $F_{q^{t}}$ be a t-extension field of $F_{q}$. For any $\alpha \in F_{q^{t}}$, the trace function $Tr_{F_{q^{t}}/F_{q}}:F_{q^{t}}\to F_{q}$ of α is defined as $Tr_{F_{q^{t}}/F_{q}}\left(\alpha \right)=\alpha +\alpha^{q}+\alpha^{q^{2}}+\cdots +\alpha^{q^{t-1}}$.*

For ease of notation, we omit the subscript $F_{q^{t}}/F_{q}$ of the trace function when it is clear from the context.

**Lemma** **1** ([19])**.** *The trace function $Tr:F_{q^{t}}\to F_{q}$ satisfies*

*(i)*  *Tr(α+β)=Tr(α)+Tr(β), $\forall \alpha ,\beta \in F_{q^{t}}$;*
*(ii)*  *Tr(aα)=aTr(α), $\forall a\in F_{q},\;\alpha \in F_{q^{t}}$.*

Since $F_{q^{t}}$ can be regarded as a *t*-dimensional vector space over $F_{q}$, let $\left\{u_{1},u_{2},\cdots ,u_{t}\right\}$ be an $F_{q}$-basis for $F_{q^{t}}$. There must exist a dual basis $\left\{{\tilde{u}}_{1},{\tilde{u}}_{2},\cdots ,{\tilde{u}}_{t}\right\}$ for $F_{q^{t}}$ such that $Tr(u_{i}{\tilde{u}}_{j})=1$ for i=j, and $Tr(u_{i}{\tilde{u}}_{j})=0$, otherwise. Furthermore, for any $\alpha \in F_{q^{t}}$, we have

(3) $$\alpha =\sum_{i\in [t]}Tr\left(\alpha u_{i}\right){\tilde{u}}_{i}.$$

Let $\alpha \in F_{q^{t}}$, g(x) be a nontrivial polynomial over $F_{q}\left[x\right]$. If g(α)=0, then α is referred to as algebraic over $F_{q}$.

**Definition** **3.** *Let 𝒞 be a linear code of length n over $F_{q}$. Then the L-interleaved code of 𝒞 is defined as*

$${𝒞}^{\left(L\right)}=\left\{{\left(c_{1},c_{2},\cdots ,c_{L}\right)}^{T}:c_{i}\in 𝒞,\;\forall i\in \left[L\right]\right\},$$

*where $c_{i}\in F_{q}^{n\times 1}$.*

## 4. SDMM Schemes with X Colluding Servers

In this section, all servers are considered untrustworthy, meaning they do not necessarily adhere faithfully to the pre-agreed protocol. We first focus on the one-sided SDMM scheme, where *N* is publicly available to all servers, and the user aims to securely compute MN without disclosing any information about *M* to any *X* colluding servers. Subsequently, we refine this to develop a fully *X*-privacy SDMM scheme.

Throughout this section, let $B=\left\{\beta_{1},\beta_{2},\cdots ,\beta_{L}\right\}\subset F_{q^{t}}$ be a set of distinct algebraic elements of degree *t* over $F_{q}$, and let $A=\left\{\alpha_{1},\alpha_{2},\cdots ,\alpha_{d}\right\}\subseteq F_{q}$. Additionally, let $U=\{u_{1},u_{2},\cdots ,u_{t}\}$ be an $F_{q}$-basis of $F_{q^{t}}$ and $\tilde{U}=\left\{{\tilde{u}}_{1},{\tilde{u}}_{2},\cdots ,{\tilde{u}}_{t}\right\}$ be the corresponding dual basis.

### 4.1. One-Sided X-Privacy SDMM Scheme

**Theorem** **1.** *Algorithm 1 is a one-sided X-privacy SDMM scheme with a download cost of $dLac\log_{2}q$ bits.*

**Algorithm 1** One-sided SDMM scheme with *X* colluding serversLet d≥L+X+t−1. The matrix $N\in F_{q^{t}}^{b\times c}$ is available to all servers, whereas $M\in F_{q^{t}}^{a\times b}$ is exclusively owned by the user. • *Upload phase*: The user partitions the matrix *M* by columns into *L* blocks of equal size as $M=\left[\begin{array}{cccc} M_{1} & M_{2} & \cdots & M_{L} \end{array}\right]$. Then, the user selects a random polynomial
(4)$$f\left(x\right)=f_{0}+f_{1}x+\cdots +f_{L+X-1}x^{L+X-1}\in F_{q^{t}}\left[x\right]$$
such that $f\left(\beta_{i}\right)=M_{i}$ for i∈[L], and calculates $f(\alpha_{j})$ to send to server *j*, where j∈[d]. • *Download phase*: The user downloads $E_{j}\left(f\left(\alpha_{j}\right)\right)$ from server *j*, where j∈[d] and
$$\begin{array}{c} E_{j}\left(f\left(\alpha_{j}\right)\right)={\left(Tr\;\left(\;\frac{f\left(\alpha_{j}\right)N_{i}\prod_{m\in [d]}\left(\beta_{i}-\alpha_{m}\right)}{\alpha_{j}-\beta_{i}}\;\right)\right)}_{i\in [L]}. \end{array}$$ • *Decoding phase*: The user chooses $h_{i,\delta }\left(x\right)\in F_{q}\left[x\right]$ as in (6). Then, MN can be decoded as
(5)$$MN=-\sum_{\delta \in [t]}\sum_{j\in [d]}{\tilde{u}}_{\delta }h_{[L],\delta }\left(\alpha_{j}\right)v_{j}^{\prime }\cdot E_{j}\left(f\left(\alpha_{j}\right)\right),$$
where $v_{j}^{\prime }=\prod_{m\in [d],m\neq j}{\left(\alpha_{j}-\alpha_{m}\right)}^{-1}$.

**Proof.**  Since *N* is a public matrix, servers partition *N* by rows into *L* blocks of equal size, so that $N={\left[\begin{array}{cccc} N_{1}^{T} & N_{2}^{T} & \cdots & N_{L}^{T} \end{array}\right]}^{T}$. Note that $\beta_{i},\;i\in \left[L\right]$ is an algebraic with degree *t* over $F_{q}$, then there must exist $h_{i,\delta }\left(x\right)\in F_{q}\left[x\right]$ such that:

(6) $$h_{i,\delta }\left(\beta_{i}\right)=u_{\delta },$$

where δ∈[t] and $\deg(h_{i,\delta }\left(x\right))\leq t-1$.

• *Correctness of Algorithm 1*:

Let 𝒞=RS(B∪A,L+X) be an RS code over $F_{q^{t}}$. Then $(f\left(\beta_{1}\right),\cdots ,f\left(\beta_{L}\right),f\left(\alpha_{1}\right),\cdots ,f\left(\alpha_{d}\right))\in 𝒞$, where f(x) is a random polynomial that satisfies (4). Consider the dual code of 𝒞:

$$\begin{array}{cc} {𝒞}^{⊥}=\{(v_{1}g\left(\beta_{1}\right),\cdots ,v_{L}g\left(\beta_{L}\right),v_{L+1}g\left(\alpha_{1}\right),\cdots , & v_{L+d}g\left(\alpha_{d}\right)):\\ \; & g\left(x\right)\in F_{q^{t}}\left[x\right],\;\deg\left(g\left(x\right)\right)<d-X\}, \end{array}$$

where $v_{i}=\prod_{m\in [L],m\neq i}{\left(\beta_{i}-\beta_{m}\right)}^{-1}\prod_{m\in [d]}{\left(\beta_{i}-\alpha_{m}\right)}^{-1}$ for i∈[L], and $v_{L+j}=\prod_{m\in [L]}{\left(\alpha_{j}-\beta_{m}\right)}^{-1}\prod_{m\in [d],m\neq j}{\left(\alpha_{j}-\alpha_{m}\right)}^{-1}$ for j∈[d]. By (2) and (4), we have

(7) $$\sum_{i\in [L]}f\left(\beta_{i}\right)v_{i}g\left(\beta_{i}\right)\;=\;\sum_{i\in [L]}M_{i}v_{i}g\left(\beta_{i}\right)\;=\;-\;\sum_{j\in [d]}f\left(\alpha_{j}\right)v_{L+j}g\left(\alpha_{j}\right).$$

Now let $g_{\delta }\left(x\right)\in F_{q^{t}}\left[x\right]$ be such that $g_{\delta }\left(\beta_{i}\right)=v_{i}^{-1}u_{\delta }N_{i}$ for i∈[L] and δ∈[t]. By Lagrange interpolation, $g_{\delta }\left(x\right)$ can be expressed as follows:

$$g_{\delta }\left(x\right)=\sum_{i\in [L]}v_{i}^{-1}u_{\delta }N_{i}\prod_{m\in [L],m\neq i}\frac{\left(x-\beta_{m}\right)}{\left(\beta_{i}-\beta_{m}\right)}\overset{\left(a\right)}{=}\sum_{i=1}^{L}v_{i}^{-1}h_{i,\delta }\left(x\right)N_{i}\prod_{m\in [L],m\neq i}\frac{\left(x-\beta_{m}\right)}{\left(\beta_{i}-\beta_{m}\right)},$$

where (a) follows from (6). Since $\deg(g_{\delta }\left(x\right))<L+t-1\leq L+d-L-T$, we have

(8) $$\left(v_{1}g_{\delta }\left(\beta_{1}\right),\cdots ,v_{L}g_{\delta }\left(\beta_{L}\right),v_{L+1}g_{\delta }\left(\alpha_{1}\right),\cdots ,v_{L+d}g_{\delta }\left(\alpha_{d}\right)\right)\in {𝒞}^{⊥}.$$

By combining (7) and (8) and then applying a trace function to both sides of the resulting equation, we obtain the following:

(9) $$\begin{array}{cc} Tr\left(u_{\delta }\sum_{i\in [L]}M_{i}N_{i}\right) & =-\sum_{j\in [d]}Tr\left(f\left(\alpha_{j}\right)v_{L+j}g_{\delta }\left(\alpha_{j}\right)\right)\\ \; & =-\sum_{j\in [d]}\;Tr\left(\;f\left(\alpha_{j}\right)v_{L+j}\sum_{i\in [L]}v_{i}^{-1}h_{i,\delta }\left(\alpha_{j}\right)N_{i}\prod_{m\in [L],\;m\neq i}\frac{\left(\alpha_{j}\;-\;\beta_{m}\right)}{\left(\beta_{i}\;-\;\beta_{m}\right)}\;\right)\\ \; & =-\sum_{j\in [d]}\sum_{i\in [L]}h_{i,\delta }\left(\alpha_{j}\right)v_{j}^{\prime }Tr\left(\frac{f\left(\alpha_{j}\right)N_{i}\prod_{m\in [d]}\left(\beta_{i}\;-\;\alpha_{m}\right)}{\alpha_{j}\;-\;\beta_{i}}\;\right). \end{array}$$

The last equality holds because $h_{i,\delta }\left(\alpha_{j}\right)\in F_{q}$ and $v_{j}^{\prime }=\prod_{m\in [d],m\neq j}{\left(\alpha_{j}-\alpha_{m}\right)}^{-1}\in F_{q}$. Hence, the user can obtain $\left\{Tr\left(u_{\delta }MN\right),\;\delta \in \left[t\right]\right\}$ by utilizing acL symbols over $F_{q}$ sent by server *j*:

$$E_{j}\left(f\left(\alpha_{j}\right)\right)={\left(Tr\left(\frac{f\left(\alpha_{j}\right)N_{i}\prod_{m\in [d]}\left(\beta_{i}-\alpha_{m}\right)}{\alpha_{j}-\beta_{i}}\;\right)\right)}_{i\in [L]}.$$

Using (3), the user can obtain MN directly as follows:

(10) $$MN=\sum_{\delta \in [t]}{\tilde{u}}_{\delta }Tr\left(u_{\delta }MN\right)=-\sum_{\delta \in [t]}\sum_{j\in [d]}{\tilde{u}}_{\delta }h_{[L],\delta }\left(\alpha_{j}\right)v_{j}^{\prime }\cdot E_{j}\left(f\left(\alpha_{j}\right)\right),$$

where $h_{[L],\delta }\left(\alpha_{j}\right)=\left(h_{1,\delta }\left(\alpha_{j}\right),h_{2,\delta }\left(\alpha_{j}\right),\cdots ,h_{L,\delta }\left(\alpha_{j}\right)\right)$ and “·” denotes the inner product. The download cost is $dLac\log_{2}q$.

• *One-sided X-privacy of Algorithm 1*: Suppose the *X* colluding servers $\left\{j_{1},j_{2},\cdots ,j_{X}\right\}$ share the encoded pieces they each received from the user, with the aim of obtaining any information about *M* from the set $m_{𝒳}=\left\{m_{j_{1}},m_{j_{2}},\cdots ,m_{j_{X}}\right\}$. Note that $m_{j_{\ell }}$ is the evaluation of a random polynomial $f\left(x\right)=f_{0}+f_{1}x+\cdots +f_{L+X-1}x^{L+X-1}\in F_{q^{t}}\left[x\right]$ at point $\alpha_{j_{\ell }}$, where ℓ∈[X] and $j_{\ell }\in \left[d\right]$. For any $\left[\begin{array}{cccc} M_{1}^{\prime } & M_{2}^{\prime } & \cdots & M_{L}^{\prime } \end{array}\right]\in F_{q^{t}}^{a\times b}$, one can obtain a unique $f^{\prime }\left(x\right)\in F_{q^{t}}\left[x\right]$ with degree L+X−1 by using the Lagrange interpolation formula such that $f^{\prime }\left(\beta_{i}\right)=M_{i}^{\prime }$ for i∈[L] and $f^{\prime }\left(\alpha_{j_{\ell }}\right)=m_{j_{\ell }}$ for ℓ∈[X]. This implies that the colluding servers learn nothing about $f\left(\beta_{i}\right)=M_{i}$ for i∈[L], i.e.,

$$I\left(M;m_{𝒳}\right)=H\left(M\right)-H\left(M|m_{𝒳}\right)\overset{\left(a\right)}{=}H\left(M\right)-H\left(M\right)=0,$$

where (a) follows from the fact that $m_{𝒳}$ cannot determine any element in *M*. □

**Remark** **1.** *According to Algorithm 1, upon receiving $E_{j}\left(f\left(\alpha_{j}\right)\right)$ from all servers, the user can directly derive MN by performing the operations as in *(10)*. However, in FTP codes, L−1 additional addition operations are required after the trace operation to obtain MN. This implies a reduced decoding delay within our scheme.*

**Example** **1.** *Here, we present a toy example of Algorithm 1 with q=11,L=2,X=6,d=11, and t=4. Therefore, we operate over $F_{11^{4}}$ (indeed, it suffices for the base field to be $F_{q}$ provided that q≥11). Let β be an algebraic with degree of *4* over $F_{11}$ such that $\beta^{4}+8\beta^{2}+10\beta +2=0$. Then $U=\{1,\beta ,\beta^{2},\beta^{3}\}$ forms an $F_{11}$-basis for $F_{11^{4}}$, and the dual basis of U is $\tilde{U}=\left\{8\beta^{3}+3\beta^{2}+9\beta +9,8\beta^{3}+\beta^{2}+\beta +9,9\beta^{2}+\beta +3,4\beta^{3}+8\beta +8\right\}$. Let $B=\{\beta ,\beta^{2}\}$ be a set of two distinct algebraic elements of degree *4* over $F_{11}$, and $A=\left\{0,1,\cdots ,10\right\}\subseteq F_{11}$. Let the public matrix $N=\left[\begin{array}{cc} \beta^{787} & \beta^{7636}\\ \beta^{9799} & \beta^{13719} \end{array}\right]\in F_{11^{4}}^{2\times 2}$.*

- Upload phase*: Let the private matrix that the user possesses be $M=\left[\begin{array}{cc} \beta^{8760} & \beta^{1520} \end{array}\right]\in F_{11^{4}}^{1\times 2}$. Since L=2, the user selects a random polynomial $f\left(x\right)=\beta^{11654}+\beta^{1332}x+\beta^{5327}x^{2}+\beta^{9564}x^{3}+\beta^{11930}x^{4}+\beta^{8951}x^{5}+\beta^{1829}x^{6}+\beta^{5462}x^{7}$, such that $f\left(\beta \right)=\beta^{8760}=M_{1},\;f\left(\beta^{2}\right)=\beta^{1520}=M_{2}$. Then the user sends $f\left(\alpha_{j}\right),\;\alpha_{j}\in A$ to server j. After receiving $f(\alpha_{j})$, server j computes $E_{j}\left(f\left(\alpha_{j}\right)\right)=\left(Tr\left(f\left(\alpha_{j}\right)N_{1}\gamma_{1,j}\right),\;Tr\left(f\left(\alpha_{j}\right)N_{2}\gamma_{2,j}\right)\right)$, where $N={\left[\begin{array}{cc} N_{1}^{T} & N_{2}^{T} \end{array}\right]}^{T}$ and $\gamma_{i,j}=\frac{\prod_{m\in [d]}\left(\beta_{i}-\alpha_{m}\right)}{\alpha_{j}-\beta_{i}}$, i∈[2].*
- Download phase*: The user downloads $E_{j}\left(f\left(\alpha_{j}\right)\right)$ from all servers.*
- Decoding phase*: The user chooses *8* polynomials over $F_{11}$:*

$$\begin{array}{cccc} h_{1,1}\left(x\right)=1, & h_{1,2}\left(x\right)=x, & h_{1,3}\left(x\right)=x^{2}, & h_{1,4}\left(x\right)=x^{3},\\ h_{2,1}\left(x\right)=1, & h_{2,2}\left(x\right)=x^{2}+8x+2, & h_{2,3}\left(x\right)=x, & h_{2,4}\left(x\right)=x^{3}+8x^{2}+2x, \end{array}$$

*such that $h_{i,\delta }\left(\beta^{i}\right)=\beta^{\delta -1}$ for i∈[2] and δ∈[4]. In summary, the user possesses the pre-computed values $v_{j}^{\prime }h_{[L],\delta }\left(\alpha_{j}\right)$ and the received values $E_{j}\left(f\left(\alpha_{j}\right)\right)$ from server j, as shown in Table 2. By *(9)*, the user obtains *4* different traces: $Tr\left(MN\right)=\left[9,9\right],\;Tr\left(\beta MN\right)=\left[7,7\right],\;Tr\left(\beta^{2}MN\right)=\left[6,0\right]$, and $Tr\left(\beta^{3}MN\right)=\left[3,2\right]$. Using the dual basis $\tilde{U}$, the user obtains the desired value:*

$$\;MN=\sum_{\delta \in [4]}{\tilde{u}}_{\delta }Tr\left(\beta^{\delta -1}MN\right)=\left[\begin{array}{cc} \beta^{7602} & \beta^{1438} \end{array}\right].$$

*In this scenario (L=2,X=6), as shown in Table 2, the user downloads *44* symbols $E_{j}\left(f\left(\alpha_{j}\right)\right)$ from the *11* servers over $F_{11}$. This is fewer than the *64* symbols typically required in conventional methods and the *54* symbols required in Scheme 2 in [18].*

### 4.2. Fully X-Privacy SDMM Scheme

In this subsection, a fully *X*-privacy SDMM scheme is proposed. This scheme ensures information-theoretic privacy for both *M* and *N* amidst possible collusion of *X* servers by choosing two random polynomials m(x) and n(x).

**Theorem** **2.** *Algorithm 2 is a fully X-privacy SDMM scheme with download cost $dLac\log_{2}q$ bits.*

**Algorithm 2** Fully SDMM scheme with *X* colluding serversLet d≥2L+2X+t−2. In this model, both $M\in F_{q^{t}}^{a\times b}$ and $N\in F_{q^{t}}^{b\times c}$ are exclusively owned by the user. • *Upload phase*: The user partitions matrix *M* by columns and *N* by rows into *L* blocks as $M=\left[\begin{array}{cccc} M_{1} & M_{2} & \cdots & M_{L} \end{array}\right]$ and $N={\left[\begin{array}{cccc} N_{1}^{T} & N_{2}^{T} & \cdots & N_{L}^{T} \end{array}\right]}^{T}$. Then the user select two random polynomials
$$\begin{array}{cc} m(x) & =m_{0}+m_{1}x+\cdots +m_{L+X-1}x^{L+X-1}\in F_{q^{t}}\left[x\right],\\ n(x) & =n_{0}+n_{1}x+\cdots +n_{L+X-1}x^{L+X-1}\in F_{q^{t}}\left[x\right], \end{array}$$
such that $m\left(\beta_{i}\right)=M_{i},\;n\left(\beta_{i}\right)=N_{i},\;i\in \left[L\right]$. The user then calculates $m\left(\alpha_{j}\right),\;n\left(\alpha_{j}\right)$ and sends these to server *j*, for j∈[d]. • *Download phase*: The user downloads $E_{j}\left(m\left(\alpha_{j}\right),n\left(\alpha_{j}\right)\right)$ from server *j*, where j∈[d] and
$$E_{j}\left(m\left(\alpha_{j}\right),n\left(\alpha_{j}\right)\right)={\left(Tr\left(\frac{m\left(\alpha_{j}\right)n\left(\alpha_{j}\right)\prod_{m\in [d]}\left(\beta_{i}-\alpha_{m}\right)}{\left(\alpha_{j}-\beta_{i}\right)}\right)\right)}_{i\in [L]}.$$ • *Decoding phase*: The user chooses $h_{i,\delta }\left(x\right)\in F_{q}\left[x\right]$ as in (6). Then MN can be decoded as follows:$$MN=-\sum_{\delta \in [t]}\sum_{j\in [d]}{\tilde{u}}_{\delta }h_{[L],\delta }\left(\alpha_{j}\right)v_{j}^{\prime }\cdot E_{j}\left(m\left(\alpha_{j}\right),n\left(\alpha_{j}\right)\right),$$
where $v_{j}^{\prime }=\prod_{m\in [d],m\neq j}{\left(\alpha_{j}-\alpha_{m}\right)}^{-1}$.

**Proof.**  The proofs of correctness and privacy closely resemble those of Theorem 1, except that f(x) is replaced by m(x)n(x) in Algorithm 2. □

## 5. SDMM Schemes with Byzantine Servers

This section focuses on the robustness of *X*-privacy SDMM schemes against Byzantine servers and stragglers. The main idea is to utilize a novel parity-check polynomial, employing the same technique described in [20], to ensure that the data returned by servers form an *L*-interleaved code of an RS code. Hence, tools from coding theory can be used to correct the errors caused by Byzantine servers and erasures caused by stragglers. Notably, our scheme does not necessitate any additional servers to handle Byzantine and straggling servers.

Throughout this section, let $B=\{\beta_{1},\beta_{2},\cdots ,\beta_{L}\}$ and $A=\{\alpha_{1},\alpha_{2},\cdots ,\alpha_{d}\}$ be two distinct public sets over $F_{q^{t}}$ and consider the RS code

$$𝒞=RS\left(B\cup A,k\right)=\left\{{\left(h(\gamma )\right)}_{\gamma \in B\cup A}:h\left(x\right)\in F_{q^{t}}\left[x\right],\deg\left(h\left(x\right)\right)<k\right\}$$

and its dual code ${𝒞}^{⊥}\;=\;GRS\left(B\cup A,L+d-k,V\right)$. Moreover, we impose the condition $d\geq q^{t-1}+k-1$ for further analysis. To enhance the robustness of the SDMM scheme against Byzantine servers, we define the parity-check polynomial of 𝒞 as

(11) $$g_{\delta }\left(x\right)=\sum_{i\in [L]}v_{i}^{-1}\frac{Tr\left(u_{\delta }\left(x-\beta_{i}\right)\right)}{\left(x-\beta_{i}\right)}k_{i}\prod_{m\in [L],m\neq i}\frac{\left(x-\beta_{m}\right)}{\left(\beta_{i}-\beta_{m}\right)},$$

where δ∈[t] and $v_{i}$ is defined in (1). Observe that $g_{\delta }\left(\beta_{i}\right)=u_{\delta }v_{i}^{-1}k_{i}$ for i∈[L], δ∈[t] and $\deg\left(g_{\delta }\left(x\right)\right)=q^{t-1}+L-2$. From $d\geq q^{t-1}+k-1$ and (2), we obtain

(12) $$\sum_{i\in \left[L\right]}\;h\left(\beta_{i}\right)v_{i}g_{\delta }\left(\beta_{i}\right)\;=\;u_{\delta }\sum_{i\in [L]}\;h\left(\beta_{i}\right)k_{i}\;=\;-\sum_{j\in [d]}\;h\left(\alpha_{j}\right)v_{L+j}g_{\delta }\left(\alpha_{j}\right).$$

**Remark** **2.** *In the scenario where h(x)=f(x) corresponds to the random polynomial in Algorithm 1, with $k_{i}=N_{i}$ for i∈[L], the scheme ensures one-sided X-privacy. On the other hand, if h(x)=m(x)n(x) represents the product of random polynomials in Algorithm 2, with $k_{i}=1$ for i∈[L], the scheme becomes a fully X-privacy SDMM scheme.*

By Remark 2 and applying a trace function to the both sides of (12), we have the following:

(13) $$Tr\left(u_{\delta }MN\right)\;=\;-\;\sum_{j\in [d]}\sum_{i\in [L]}Tr\left(u_{\delta }\left(\alpha_{j}\;-\;\beta_{i}\right)\right)s_{j,i},$$

where $s_{j,i}=Tr\left(\frac{h\left(\alpha_{j}\right)v_{L+j}v_{i}^{-1}k_{i}}{\left(\alpha_{j}-\beta_{i}\right)}\prod_{m\in [L],m\neq i}\frac{\left(\alpha_{j}-\beta_{m}\right)}{\left(\beta_{i}-\beta_{m}\right)}\right)$.

From (3) and (13), it is evident that the user can obtain MN after receiving $ℛ≜{\left[\begin{array}{cccc} s_{1}^{T} & s_{2}^{T} & \cdots & s_{d}^{T} \end{array}\right]}^{T}$, where $s_{j}=\left[\begin{array}{cccc} s_{j,1} & s_{j,2} & \cdots & s_{j,L} \end{array}\right]\in F_{q}^{a\times cL}$ represents the data sent by server j,j∈[d] to the user, and $s_{j,i}\in F_{q}^{a\times c}$ for all i∈[L]. To ensure that our scheme provides *X*-privacy while also being robust against Byzantine servers and resilient to stragglers, additional conditions are necessary so that the data from all servers forms an *L*-interleaved code. In other words, because the data transmitted by the servers forms an *L*-interleaved code, tools from coding theory can be used to correct errors introduced by Byzantine servers and handle erasures caused by stragglers. Specifically, for any i∈[L], $\tau_{i}≜\left(s_{1,i},s_{2,i},\cdots ,s_{d,i}\right)$ is a codeword of an *L*-interleaved RS code. Since $s_{j,i}\in F_{q}^{a\times c}$, according to the definition of *L*-interleaved codes, let $s_{j,i}^{\left(\ell_{1},\ell_{2}\right)}$ be the element in the $\ell_{1}$-th row and $\ell_{2}$-th column of $s_{j,i}$, where $\forall \ell_{1}\in \left[a\right]$ and $\forall \ell_{2}\in \left[c\right]$. Then $\left(s_{1,i}^{\left(\ell_{1},\ell_{2}\right)},s_{2,i}^{\left(\ell_{1},\ell_{2}\right)},\cdots ,s_{d,i}^{\left(\ell_{1},\ell_{2}\right)}\right)$ forms a codeword of an RS code.

**Figure 2:**
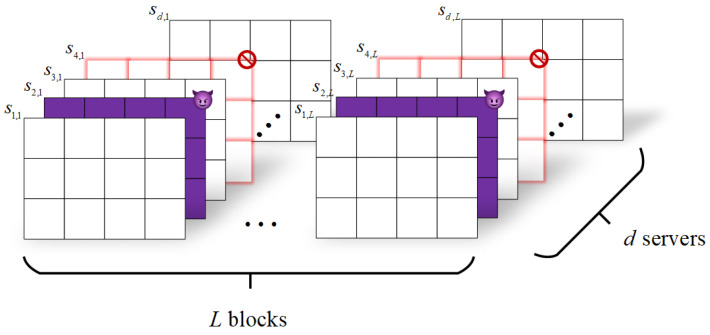
The responses from all *d* servers. Response from server j,j∈[d] consists of *L* matrices, depicted in the figure as $s_{j,1},s_{j,2},\cdots ,s_{j,L}$. Byzantine server is represented by the purple layer, and straggler by the blurred red layer. For any i∈[L], $\tau_{i}≜\left(s_{1,i},s_{2,i},\cdots ,s_{d,i}\right)$ forms a codeword of an *L*-interleaved RS code.

Figure 2 illustrates the responses from all servers in our scheme. As described above, each horizontal layer in Figure 2, $s_{j}=\left[\begin{array}{cccc} s_{j,1} & s_{j,2} & \cdots & s_{j,L} \end{array}\right]$, represents the response from server *j*. After satisfying the necessary constraints, each vertical column in Figure 2, $\left(s_{1,i},s_{2,i},\cdots ,s_{d,i}\right)$, forms a codeword in an *L*-interleaved RS code. In Figure 2, we use a purple layer to indicate that a server has returned incorrect data. For instance, server 2 in the figure returns $s_{2,i}+z_{2,i}$, where $z_{2,i}$ is a non-zero matrix. Consequently, the second symbol in the codeword $\tau_{i}$ is treated as an error. If a server fails to respond promptly, we use a blurred red layer to represent this server, as seen with server 4 in Figure 2, resulting in the fourth symbol in the codeword $\tau_{i}$ being treated as an erasure. The user can then utilize linear coding methods to address these errors and erasures to obtain $\tau_{i}$, and subsequently use Equation (13) to derive MN.

For the sake of clarity, we define $\tau_{i}≜\left(s_{1,i},s_{2,i},\cdots ,s_{d,i}\right)\in F_{q}^{1\times d}$. It should be noted that this definition does not impact our proof, as when $s_{j}$ is reshaped into a vector of length acL in $F_{q}$, and $s_{j,i}$ becomes an element in $F_{q}$. Subsequently, we will demonstrate how to operate ℛ to transform it into a codeword of an *L*-interleaved code of an RS code. Hence, the enhanced SDMM schemes possess security against Byzantine servers and the influence caused by stragglers. The results can be summarized by the following theorem.

**Theorem** **3.** *For a fixed i,i∈[L], consider $\tau_{i}=\left(s_{1,i},s_{2,i},\cdots ,s_{d,i}\right)$:*

*(i)*  *If $B\cup A=F_{q^{t}}$ and d≥Δ, then the minimum weight of $\tau_{i}$ is greater than d−Δ, where $\Delta =\left(k+L-2\right)q^{t-1}$ and ${\left({\left(\alpha_{j}-\beta_{i}\right)}^{q^{t-1}}s_{j,i}\right)}_{j\in [d]}\in RS\left(A,\Delta +1\right)$;*
*(ii)*  *If $k\leq d-2q^{t-1}+1$, then $\tau_{i}\in GRS\left(A,d-2\ell +1,V\right)$ for some V, where $\ell =\lceil \frac{d-k+2}{2q^{t-1}}\rceil -1$.*

### 5.1. Enhanced SDMM Scheme with Full-Length RS Code

In this subsection, we let $B\cup A=F_{q^{t}}$, thus V=(1,1,⋯,1). By (13), the user can obtain $\left\{Tr\left(u_{\delta }MN\right),\;\delta \in \left[t\right]\right\}$ by receiving acL symbols over $F_{q}$ from server *j*:

$$s_{j}={\left({\left(s_{j,i}\right)}_{i\in [L]}\right)}^{T}={\left({\left(Tr\left(\frac{h\left(\alpha_{j}\right)\nu_{j}C_{i}}{\left(\alpha_{j}-\beta_{i}\right)}\right)\right)}_{i\in [L]}\right)}^{T},$$

where $\nu_{j}=\prod_{m\in [L],m\neq i}\left(\alpha_{j}-\beta_{m}\right)$ and $C_{i}=k_{i}\prod_{m\in [L],m\neq i}$${\left(\beta_{i}-\beta_{m}\right)}^{-1}$.

**Proposition** **1.** *For a fixed i,i∈[L], if d≥Δ, then the minimum weight of $\tau_{i}$ is greater than d−Δ.*

**Proof.**  Note that ${\left({\left(\alpha_{j}-\beta_{i}\right)}^{q^{t-1}}s_{j,i}\right)}_{j\in [d]}$ can be represented as

$$\begin{array}{cc} {\left(F_{i}\left(\alpha_{j}\right)\right)}_{j\in [d]} & ={\left({\left(\alpha_{j}-\beta_{i}\right)}^{q^{t-1}}Tr\left(\frac{h\left(\alpha_{j}\right)\nu_{j}C_{i}}{\left(\alpha_{j}-\beta_{i}\right)}\right)\right)}_{j\in [d]}\\ \; & ={\left({\left(\alpha_{j}-\beta_{i}\right)}^{q^{t-1}}\sum_{\delta \in [t]}{\left(\frac{h\left(\alpha_{j}\right)\nu_{j}C_{i})}{\left(\alpha_{j}-\beta_{i}\right)}\right)}^{q^{\delta -1}}\right)}_{j\in [d]}\\ \; & ={\left({\left(\alpha_{j}-\beta_{i}\right)}^{q^{t-1}-1}h\left(\alpha_{j}\right)\nu_{j}C_{i}+\cdots +{\left(h\left(\alpha_{j}\right)\nu_{j}C_{i}\right)}^{q^{t-1}}\right)}_{j\in [d]}. \end{array}$$

Hence,

$$F_{i}\left(x\right)={\left(x-\beta_{i}\right)}^{q^{t-1}-1}h\left(x\right)\prod_{m\in [L],m\neq i}\left(x-\beta_{i}\right)C_{i}+\cdots +{\left(h\left(x\right)\prod_{m\in [L],m\neq i}\left(x-\beta_{i}\right)C_{i}\right)}^{q^{t-1}}$$

is a polynomial with degree $\Delta =\max\left\{q^{t-1}+k+L-3,\cdots ,\left(k+L-2\right)q^{t-1}\right\}$. Then

$${\left({\left(\alpha_{j}-\beta_{i}\right)}^{q^{t-1}}s_{j,i}\right)}_{j\in [d]}\in RS\left(A,\Delta +1\right).$$

This completes the proof of Theorem 3 (i). □

### 5.2. Enhanced SDMM Scheme with Non-Full-Length RS Code

Proposition 1 applies only when k<q−L+2 and requires a full-length RS code. In this subsection, we study the properties of $\tau_{i}$ when *k* is large. For a non-full-length RS code, the $s_{j,i}$ in (13) is equal to $Tr\left(\frac{h\left(\alpha_{j}\right)\nu_{j}^{\prime }C_{i}^{\prime }}{{\left(\alpha_{j}\;-\;\beta_{i}\right)}^{2}}\right)$, where $\nu_{j}^{\prime }=\prod_{m\in [d],m\neq j}{\left(\alpha_{j}-\alpha_{m}\right)}^{-1}$ and $C_{i}^{\prime }=k_{i}\prod_{m\in [d]}$$\left(\beta_{i}-\alpha_{m}\right)$.

**Proposition** **2.** *For a fixed i,i∈[L], $\tau_{i}=\left(s_{1,i},s_{2,i},\cdots ,s_{d,i}\right)$ is a codeword of GRS(A,d−2ℓ+1,V) for some V.*

**Proof.**  Note that $\left(h\left(\alpha_{1}\right),h\left(\alpha_{2}\right),\cdots ,h\left(\alpha_{d}\right)\right)\in RS\left(A,k\right)$. Let $g_{\delta ,i}^{\left(\ell \right)}\left(x\right)=\frac{Tr\left(u_{\delta }{\left(x-\beta_{i}\right)}^{2\ell }\right)C_{i}^{\prime }}{{\left(x-\beta_{i}\right)}^{2}}$, and let *ℓ* be an integer such that $1\leq \ell <\frac{d-k+2}{2q^{t-1}}$. Since $\deg\left(g_{\delta ,i}^{\left(\ell \right)}\left(x\right)\right)=2\ell q^{t-1}-2<d-k$ and (2), we have

$$\begin{array}{cc} \sum_{j\in [d]}Tr\left(h\left(\alpha_{j}\right)\nu_{j}^{\prime }g_{\delta ,i}^{\left(\ell \right)}\left(\alpha_{j}\right)\right) & \;=\;\sum_{j\in [d]}Tr\left(Tr\left(u_{\delta }{\left(\alpha_{j}-\beta_{i}\right)}^{2\ell }\right)\frac{h\left(\alpha_{j}\right)\nu_{j}^{\prime }C_{i}^{\prime }}{{\left(\alpha_{j}-\beta_{i}\right)}^{2}}\right)\\ \; & \;=\;\sum_{j\in [d]}Tr\left(\;u_{\delta }{\left(\alpha_{j}\;-\;\beta_{i}\right)}^{2\ell }Tr\left(\frac{h\left(\alpha_{j}\right)\nu_{j}^{\prime }C_{i}^{\prime }}{{\left(\alpha_{j}\;-\;\beta_{i}\right)}^{2}}\right)\;\right)\;=\;\sum_{j\in [d]}Tr\left(\;u_{\delta }{\left(\alpha_{j}\;-\;\beta_{i}\right)}^{2\ell }s_{j,i}\;\right)\\ \; & =Tr\left(u_{\delta }\sum_{j\in [d]}{\left(\alpha_{j}-\beta_{i}\right)}^{2\ell }s_{j,i}\right)=0. \end{array}$$

Based on the final equality in the above equation, and (3), we have

$$\sum_{\delta \in [t]}{\tilde{u}}_{\delta }Tr\left(\;u_{\delta }\sum_{j\in [d]}{\left(\alpha_{j}-\beta_{i}\right)}^{2\ell }s_{j,i}\right)=\sum_{j\in [d]}{\left(\alpha_{j}-\beta_{i}\right)}^{2\ell }s_{j,i}=0.$$

The above equation can be Expressed in matrix form as follows:

$$\left[\begin{array}{cccc} {\left(\alpha_{1}-\beta_{i}\right)}^{2} & {\left(\alpha_{2}-\beta_{i}\right)}^{2} & \cdots & {\left(\alpha_{d}-\beta_{i}\right)}^{2}\\ {\left(\alpha_{1}-\beta_{i}\right)}^{3} & {\left(\alpha_{2}-\beta_{i}\right)}^{3} & \cdots & {\left(\alpha_{d}-\beta_{i}\right)}^{3}\\ \vdots & \vdots & \ddots & \vdots \\ {\left(\alpha_{1}-\beta_{i}\right)}^{2\ell } & {\left(\alpha_{2}-\beta_{i}\right)}^{2\ell } & \cdots & {\left(\alpha_{d}-\beta_{i}\right)}^{2\ell } \end{array}\right]\left[\begin{array}{c} s_{1,i}\\ s_{2,i}\\ \vdots \\ s_{d,i} \end{array}\right]=0,$$

for $\ell =\lceil \frac{d-k+2}{2q^{t-1}}\rceil -1$. Hence, $\tau_{i}=\left(s_{1,i},s_{2,i},\cdots ,s_{d,i}\right)\in GRS\left(A,d-2\ell +1,V\right)$ for some multiplier *V*. This completes the proof of Theorem 3 (ii). □

**Remark** **3.** *In Theorem 3 (i), since ${\left({\left(\alpha_{j}-\beta_{i}\right)}^{q^{t-1}}s_{j,i}\right)}_{i\in [d]}\in RS\left(A,\Delta +1\right)$, it is sufficient for the user to accurately obtain MN by receiving the computation results from Δ+1 normal servers. Hence, this scheme is capable of mitigating the impact of d−Δ−1 stragglers on computation time or providing security against $\left\lceil \frac{d-\Delta }{2}\right\rceil$ Byzantine servers. Similarly, in Theorem 3 (ii), this scheme can at most eliminate the influence caused by 2ℓ−1 stragglers or provide security against ℓ Byzantine servers.*

**Example** **2.** *Here, we provide an example of a one-sided X-privacy scheme over $F_{11^{2}}$ that is robust to Byzantine servers and stragglers, with parameters d=95,L=2, and X=6. Let β be algebraic with degree of *2* over $F_{11}$ such that $\beta^{2}+7\beta +2=0$, then U={1,β} be the $F_{11}$-basis for $F_{11^{2}}$ and the dual basis of U is $\tilde{U}=\left\{\beta^{113},\beta^{42}\right\}$. Let B={1,β}, $A=\{\beta^{2},\beta^{3},\cdots ,\beta^{96}\}$ and the public matrix $N=\left[\begin{array}{cc} \beta^{78} & \beta^{34}\\ \beta^{91} & \beta^{56} \end{array}\right]\in F_{11^{2}}^{2\times 2}$.*

- Upload phase*: Let the private matrix that the user possesses be $M=\left[\begin{array}{cc} \beta^{118} & \beta^{116} \end{array}\right]\in F_{11^{2}}^{1\times 2}$. Since L=2,X=6, the user selects a random polynomial with degree L+X−1 as $h\left(x\right)=\beta^{45}+\beta^{78}x+\beta^{15}x^{2}+\beta^{92}x^{3}+\beta^{33}x^{4}+\beta^{56}x^{6}+\beta^{88}x^{7}$, such that $h\left(1\right)=\beta^{118}=M_{1},\;h\left(\beta \right)=\beta^{116}=M_{2}$. Then the user sends $h\left(\alpha_{j}\right),\;\alpha_{j}\in A$ to server j. Let*
  $$g_{\delta }\left(x\right)=\frac{v_{1}^{-1}Tr\left(u_{\delta }\left(x-1\right)\right)N_{1}}{\left(x-1\right)}\frac{x-\beta }{1-\beta }+\frac{v_{2}^{-1}Tr\left(u_{\delta }\left(x-\beta \right)\right)N_{2}}{\left(x-\beta \right)}\frac{x-1}{\beta -1},$$
  *where δ∈[2]. Since $\deg(g_{\delta }\left(x\right))=11<d+L-8$, we have*
  $$\sum_{i\in [2]}h\left(\beta_{i}\right)v_{i}g_{\delta }\left(\beta_{i}\right)=u_{\delta }MN=-\sum_{j\in [95]}h\left(\alpha_{j}\right)v_{2+j}g_{\delta }\left(\alpha_{j}\right).$$
  *Apply a trace function to both sides of the above equation:*
  (14) $$\begin{array}{cc} Tr(u_{\delta }MN) & =\;-\;\sum_{j\in [95]}Tr\left(h\left(\alpha_{j}\right)v_{2+j}\sum_{i\in [2]}\left(\frac{v_{i}^{-1}Tr\left(u_{\delta }\left(\alpha_{j}\;-\;\beta_{i}\right)\right)N_{i}}{\left(\alpha_{j}\;-\;\beta_{i}\right)}\prod_{m\in [2],m\neq i}\frac{\left(\alpha_{j}\;-\;\beta_{m}\right)}{\left(\beta_{i}\;-\;\beta_{m}\right)}\right)\right)\\ \; & =\sum_{j\in [95]}\sum_{i\in [2]}Tr\left(u_{\delta }\left(\alpha_{j}-\beta_{i}\right)\right)Tr\left(\frac{h\left(\alpha_{j}\right)N_{i}}{\left(\alpha_{j}-\beta_{i}\right)}\prod_{m\in [95],m\neq j}\frac{\left(\beta_{i}-\alpha_{m}\right)}{\left(\alpha_{j}-\alpha_{m}\right)}\right)\\ \; & =-\sum_{j\in [95]}\sum_{i\in [2]}Tr\left(u_{\delta }\left(\alpha_{j}-\beta_{i}\right)\right)s_{j,i}, \end{array}$$
  *where $s_{j,i}=Tr\left(\frac{h\left(\alpha_{j}\right){\left(\prod_{m\in [95],m\neq j}\left(\alpha_{j}-\alpha_{m}\right)\right)}^{-1}N_{i}\prod_{m\in [95]}\left(\beta_{i}-\alpha_{m}\right)}{{\left(\alpha_{j}-\beta_{i}\right)}^{2}}\right)$.*
*Hence, after receiving $h(\alpha_{j})$, server j computes*
$$E_{j}\left(h\left(\alpha_{j}\right)\right)=\left(s_{j,1},s_{j,2}\right)=\left(Tr\left(\frac{h\left(\alpha_{j}\right)\nu_{j}^{\prime }C_{1}^{\prime }}{{\left(\alpha_{j}-1\right)}^{2}}\right),Tr\left(\frac{h\left(\alpha_{j}\right)\nu_{j}^{\prime }C_{2}^{\prime }}{{\left(\alpha_{j}-\beta \right)}^{2}}\right)\right),$$
  *where $\nu_{j}^{\prime }=\prod_{m\in [95],m\neq j}{\left(\alpha_{j}-\alpha_{m}\right)}^{-1},\;C_{i}^{\prime }=N_{i}\prod_{m\in [d]}\left(\beta_{i}-\alpha_{m}\right)$.*
- Download phase*: The user downloads $E_{j}\left(h\left(\alpha_{j}\right)\right)=\left(s_{j,1},s_{j,2}\right)$ from all servers.*
- Decoding phase*: When the ${\left\{E_{j}\left(h\left(\alpha_{j}\right)\right)\right\}}_{j\in [95]}$ downloaded by the user from all servers are error-free, the user can obtain Tr(MN)=[8,0] and Tr(βMN)=[2,6] by *(14)*. According to *(3)* and $\tilde{U}$, the user can derive MN as follows:*
  $$MN=\beta^{113}Tr\left(MN\right)+\beta^{42}Tr\left(\beta MN\right)=\left[\begin{array}{cc} \beta^{13} & \beta^{30} \end{array}\right].$$
  *In this scenario, our scheme has one-sided *6*-privacy. Moreover, based on the parameters provided in this example, we have:*
  (15) $$\left[\begin{array}{cccc} {\left(\beta^{82}\right)}^{2} & {\left(\beta^{41}\right)}^{2} & \cdots & 2^{2}\\ {\left(\beta^{82}\right)}^{3} & {\left(\beta^{41}\right)}^{3} & \cdots & 2^{3}\\ \vdots & \vdots & \ddots & \vdots \\ {\left(\beta^{82}\right)}^{8} & {\left(\beta^{41}\right)}^{8} & \cdots & 2^{8} \end{array}\right]\left[\begin{array}{cc} 0 & 5\\ 7 & 3\\ \vdots & \vdots \\ 7 & 9 \end{array}\right]=0,\;\left[\begin{array}{cccc} {\left(b^{56}\right)}^{2} & {\left(b^{83}\right)}^{2} & \cdots & {\left(b^{5}\right)}^{2}\\ {\left(b^{56}\right)}^{3} & {\left(b^{83}\right)}^{3} & \cdots & {\left(b^{5}\right)}^{3}\\ \vdots & \vdots & \ddots & \vdots \\ {\left(b^{56}\right)}^{8} & {\left(b^{83}\right)}^{8} & \cdots & {\left(b^{5}\right)}^{8} \end{array}\right]\left[\begin{array}{cc} 7 & 0\\ 9 & 1\\ \vdots & \vdots \\ 8 & 0 \end{array}\right]=0,$$
  *where $s_{1,1}\;=\;{\left[0,5\right]}^{T},s_{2,1}\;=\;{\left[7,3\right]}^{T},\;\cdots \;,s_{95,1}\;=\;{\left[7,9\right]}^{T}$ and $s_{1,2}\;=\;{\left[7,0\right]}^{T},s_{2,2}\;=\;{\left[9,1\right]}^{T},\;\cdots \;,s_{95,2}\;=\;{\left[8,0\right]}^{T}$ are the data sent from the servers. By *(15)*, it is evident that for a fixed i∈{1,2}, $\left(s_{1,i},s_{2,i},\cdots ,s_{95,i}\right)$ is a codeword in a *2*-interleaved code of GRS(A,88,V) for some V. Hence, according to coding theory, the scheme in this example also provides security against up to *4* Byzantine servers or resiliency against up to *7* stragglers.*

## 6. Conclusions

This paper proposes novel communication-efficient SDMM schemes that leverage trace polynomials. As investigated in Section 4, the innovative use of minimal polynomials leads to low-degree parity-check polynomials, resulting in a reduction of the requisite number of servers for the one-sided *X*-privacy SDMM scheme to d≥L+X+t−1 (d≥2L+2X+t−1 for fully *X*-privacy SDMM). Although the data transmission per server is *L* times that of previous work [18], the overall data transmitted across the network is reduced due to fewer involved servers. Furthermore, in Section 5, new parity-check polynomials are constructed to ensure that the data returned by servers form an *L*-interleaved code of an RS code. This enhancement endows our SDMM schemes with not only *X*-privacy but also security against Byzantine servers, potentially mitigating the effects of stragglers. Compared to other SDMM schemes based on trace polynomials, our proposed scheme features reduced sub-packetization and an appropriate number of servers, making it suitable for real-world applications. Additionally, this work is the first to consider the security and resiliency of SDMM schemes based on trace polynomials. Investigating the theoretical limits of the download cost in SDMM schemes, and proposing explicit SDMM schemes with lower download costs, represents a promising direction for future research.

## Figures and Tables

**Table 1:** Comparison of key parameters for different fully *X*-privacy SDMM schemes.

	Download Cost	Sub-Packetization *t*	Number of Servers *d*	Restrictions	Remark
FTP codes	$ac\sum_{i=1}^{L}N_{L}\prod_{m\in \{1,\cdots ,L\}\setminus \{i\}}p_{m}$ *	$t=p_{1}p_{2}\cdots p_{L}$	$p_{L}+2L+2X-2$	q≥d	No
Scheme 1 in [18]	$\geq ac(Lq^{t-1}+L+2X-1)$	$L+d\leq q^{t}$	$\geq Lq^{t-1}+L+2X-1$		No
Scheme 2 in [18]	$\geq ac\left(t-s\right)\left(Lq^{s}+L+2X-1\right)$	$L+d\leq q^{t}$	$\geq Lq^{s}+L+2X-1$	s<t	No
Fully SDMM scheme in this paper	≥(2L+2X+t−2)Lac	$NDA{\left(t\right)}_{F_{q}}\geq L$ **	≥2L+2X+t−2	q≥d	No
Scheme in Theorem 3 (i)	$\geq (\left(3L+2X-3\right)q^{t-1}+1)Lac$	$L+d=q^{t}$	$\geq q^{t-1}\left(3L+2X-3\right)$		Yes
Scheme in Theorem 3 (ii)	$\geq (q^{t-1}\;+\;2L\;+\;2X\;-\;2\ell \;-\;1)Lac$ ***	$L+d\leq q^{t}$	$\geq q^{t-1}+2L+2X-2$		Yes

* $N_{L}=p_{L}+2L+2T-2$; ** $NDA{\left(t\right)}_{F_{q}}$ means the number of distinct algebraic with degree *t* over $F_{q}$; *** $\ell =\lceil \frac{d-k+2}{2q^{t-1}}\rceil$.

**Table 2:** The pre-computed values and received values in Example 1.

Sever *j*	*A*	$v_{j}^{\prime }h_{[L],\delta }\left(\alpha_{j}\right)$	$E_{j}\left(f\left(\alpha_{j}\right)\right)$
1	0	[10,10],[0,9],[0,0],[0,0]	[[10,4],[4,7]]
2	1	[10,10],[10,0],[10,10],[10,0]	[[6,3],[10,4]]
3	2	[10,10],[9,0],[7,9],[3,0]	[[9,9],[8,3]]
4	3	[10,10],[8,9],[2,8],[6,5]	[[0,4],[1,9]]
5	4	[10,10],[7,5],[6,7],[2,9]	[[7,3],[3,4]]
6	5	[10,10],[6,10],[8,6],[7,6]	[[4,3],[5,3]]
7	6	[10,10],[5,2],[8,5],[4,1]	[[2,1],[7,4]]
8	7	[10,10],[4,3],[6,4],[9,10]	[[3,10],[5,9]]
9	8	[10,10],[3,2],[2,3],[5,5]	[[2,3],[4,8]]
10	9	[10,10],[2,10],[7,2],[8,2]	[[4,3],[5,10]]
11	10	[10,10],[1,5],[10,1],[1,6]	[[1,5],[8,10]]

## Data Availability

Data are contained within the article.

## References

[1] Yu Q., Maddah-Ali M.A., Avestimehr A.S. Polynomial codes: An optimal design for high-dimensional coded matrix multiplication. Proceedings of the Advances in Neural Information Processing Systems (NIPS).

[2] Chang W., Tandon R. On the capacity of secure distributed matrix multiplication. Proceedings of the 2018 IEEE Global Communications Conference (GLOBELCOM).

[3] D’Oliveira R.G., Rouayheb S.E., Karpuk D. (2020). Gasp codes for secure distributed matrix multiplication. IEEE Trans. Inf. Theory.

[4] D’Oliveira R.G., Rouayheb S.E., Heinlein D., Karpuk D. (2021). Degree tables for secure distributed matrix multiplication. IEEE J. Sel. Areas Inf. Theory.

[5] D’Oliveira R.G., Rouayheb S.E., Heinlein D., Karpuk D. Notes on communication and computation in secure distributed matrix multiplication. Proceedings of the 2020 IEEE Conference on Communications and Network Security (CNS).

[6] Yang H., Lee J. (2019). Secure distributed computing with straggling servers using polynomial codes. IEEE Trans. Inf. Forensics Secur..

[7] Kakar J., Ebadifar S., Sezgin A. (2019). On the capacity and straggler-roubustness of distributed matrix multiplication. IEEE Access.

[8] López H.H., Matthews G.L., Valvo D. Secure MatDot codes: A secure, distributed matrix multiplication scheme. Proceedings of the 2022 IEEE Information Theory Workshop (ITW).

[9] Mital N., Ling C., Gündüzm D. (2022). Secure distributed matrix computation with discrete fourier transform. IEEE Trans. Inf. Theory.

[10] Yu Q., Avestimehr A.S. Entangled polynomial codes for secure, private, and batch distributed matrix multiplication: Breaking the “cubic” barrier. Proceedings of the 2020 IEEE International Symposium on Information Theory (ISIT).

[11] Yu Q., Li S., Raviv N., Kalan S.M.M., Soltanolkotabi M., Avestimehr S.A. Lagrange coded computing: Optimal design for resiliency, security, and privacy. Proceedings of the 22nd International Conference on Artificial Intelligence and Statiscs (AISTATS).

[12] Byrne E., Gnilke O.W., Kliewer J. (2023). Straggler-and adversary-tolerant secure distributed matrix multiplication using polynomial codes. Entropy.

[13] Makkonen O., Hollanti C. (2024). General framework for linear secure distributed matrix multiplication with byzantine servers. IEEE Trans. Inf. Theory.

[14] Guruswami V., Wootters M. (2017). Repairing Reed–Solomon codes. IEEE Trans. Inf. Theory.

[15] Mardia J., Bartan B., Wootters M. (2019). Repairing multiple failures for scalar mds codes. IEEE Trans. Inf. Theory.

[16] Tamo I., Ye M., Barg A. Optimal repair of Reed–Solomon codes: Achieving the cut-set bound. Proceedings of the 2017 IEEE 58th Annual Symposium on Foundations Computer Science (FOCS).

[17] Machado R.A., D’Oliveira R.G., Rouayheb S.E., Heinlein D. Field trace polynomial codes for secure distributed matrix multiplication. Proceedings of the 2021 XVII International Symposium Problems Redundancy in Information and Control Systems (RED).

[18] Kiah H.M., Kim W., Kruglik S., Ling S., Wang H. (2024). Explicit low-bandwidth evaluation schemes for weighted sums of Reed–Solomon-coded symbols. IEEE Trans. Inf. Theory.

[19] Lidl R., Niederreiter H. (1997). Finite Filed.

[20] Kruglik S., Luo G., Lim W., Singhvi S., Kiah H.M., Ling S., Wang H. Repair of Reed–Solomon codes in the presence of erroneous nodes. Proceedings of the 2023 IEEE International Symposium on Information Theory (ISIT).

